# Management der posttraumatischen Bulbushypotonie

**DOI:** 10.1007/s00347-020-01290-4

**Published:** 2021-01-04

**Authors:** Arne Viestenz, Andrea Huth, Jens Heichel, Berthold Seitz

**Affiliations:** 1 Klinik und Poliklinik für Augenheilkunde, Universitätsklinikum Halle UKH, Martin-Luther-Universität Halle-Wittenberg, Ernst-Grube-Str. 40, 06120 Halle (Saale), Deutschland; 2 Klinik für Augenheilkunde, Universitätsklinikum des Saarlandes UKS, Kirrberger Str., Geb. 22, 66424 Homburg/Saar, Deutschland

**Keywords:** Augenverletzung, Gonioskopie, Stauungspapille e vacuo, Zyklodialyse, Zyklopexie, Eye injury, Gonioscopy, Papilledema e vacuo, Cyclodialysis, Cyclopexy

## Abstract

Die Bulbushypotonie nach Trauma (3 % nach Bulbuskontusion, 50–74 % nach offener Augenverletzung) kann schwere sekundäre Schäden des Bulbus zur Folge haben. Neben Hornhautfalten, Ziliarkörper- und Aderhautabhebung, Stauungspapille e vacuo und Makulasternfalten kann die Schrumpfung des Auges zu einer erheblichen Visusminderung führen. Konsekutiv kann das kontralaterale Auge mit einer okulären Hypertension reagieren. Die Ursache der Hypotonie muss diagnostiziert und kausal behandelt werden. Wenn mehr als 210 Grad des Ziliarkörpers verbleiben, ist ein Bulbuserhalt möglich. Häufige Ursache der posttraumatischen Hypotonie ist eine Zyklodialyse. Kleinere Zyklodialysespalten verschließen sich unter Zykloplegie, größere werden chirurgisch versorgt. Die Zyklopexie kann mit einer primären Wundversorgung oder auch einer Pol-zu-Pol-Chirurgie kombiniert werden. Alternativ sind bei persistierender Hypotonia bulbi eine Silikonölendotamponade bzw. eine Okklusion des Schlemm-Kanals möglich. Die posttraumatische Bulbushypotonie ist komplex und bedarf einer exakten Diagnostik, um ihre Ursachen differenziert und gezielt behandeln zu können.

## Abstract (en)

The posttraumatic ocular hypotony (3% after ocular contusion, 50–74% after open globe injury) can lead to severe secondary damage of the eyeball. In addition to corneal folds, ciliary body and choroidal detachment, papilledema e vacuo and macular folds, the shrinking of the eye can lead to substantial visual impairment. Subsequently, the contralateral eye may react with ocular hypertension. The cause of the hypotony must be identified and causally treated. A preservation of the globe is possible if more than 210 ° of the ciliary body are intact. One of the major causes of posttraumatic hypotony is cyclodialysis. Smaller cyclodialysis clefts respond to a cycloplegic treatment, larger clefts require a surgical approach. The direct cyclopexy can be combined with primary wound repair or pole to pole surgery. An alternative consists of silicone oil endotamponade or occlusion of Schlemm’s canal. The posttraumatic ocular hypotony is complex and requires exact diagnostics to be able to differentially and specifically treat the causes of hypotony.

## Lernziele

Nach der Lektüre dieses Beitrags …

- können Sie die posttraumatische Bulbushypotonie und deren Ursachen zuverlässig identifizieren,
- sind Sie in der Lage, die pathophysiologischen Ursachen der posttraumatischen Bulbushypotonie zu differenzieren,
- gelingt es Ihnen, die klinischen Zeichen der Bulbushypotonie einzuordnen,
- ist es Ihnen möglich, die richtige operative und medikamentöse Therapie einzuleiten.

## Einleitung

Seit dem Einzug der **Vitrektomie**

Vitrektomie

in die Ophthalmochirurgie hat sich das Management von schweren Augenverletzungen grundsätzlich verändert. Eine komplette Rekonstruktion des gesamten Auges – sozusagen von Pol zu Pol – ist Dank temporärer Keratoprothese, Fortschritten in der Hornhauttransplantation, Irisersatz und minimal-invasiver Intraokularlinsenimplantation in Kombination mit vitreoretinaler Chirurgie möglich [1, 2].

Trotz optimal durchgeführter und aufwendiger Chirurgie sowie wieder angelegter Netzhaut erblinden bis zu 19 % der schwer verletzten Augen [2, 3, 4, 5, 6]. Ein nach der Verletzung blindes Auge sollte nicht primär enukleiert werden. Insofern es noch anatomisch rekonstruiert werden kann [1, 2, 3, 4, 6, 7, 8, 9, 10], hat der Chirurg hier die Möglichkeit, im Rahmen der Wundversorgung gleich die Kammerwasserproduktionsstrukturen zu rekonstruieren oder mit einer primären Vitrektomie späteren Komplikationen vorzubeugen [11].

### Cave

Eine primäre Enukleation nach Trauma sollte vermieden werden!

Das Interesse der jungen Ophthalmochirurgen, die Techniken der **Maximalrekonstruktionschirurgie**

Maximalrekonstruktionschirurgie

zu erlernen, ist ungebrochen. Allerdings kann ein kleiner Schritt, der nicht durchgeführt wurde, eine Bulbushypotonie zur Folge haben.

## Definition der Hypotonia bulbi

Über die Definition der **Bulbushypotonie**

Bulbushypotonie

besteht keine Einigkeit, was auch an den Messfehlern der Applanationstonometrie liegen mag. Von einer Bulbushypotonie spricht man bei **intraokularen Druckwerten**

intraokularen Druckwerten

(IOD) von weniger als 6–8 mm Hg. Die intraokularen Umbauprozesse durch die Bulbushypotonie setzen meist erst bei einem IOD kleiner 4,5 mm Hg ein [6, 12, 13].

### Merke

Ein Augeninnendruck unter 4,5 mm Hg ist ein Risiko für eine Bulbusatrophie mit Schrumpfung.

Dellaporta beschrieb 1954 die mit einer Bulbushypotonie assoziierte **Makulopathie**

Makulopathie

[14]. Nicht jedes Auge entwickelt unter einem applanatorisch niedrig gemessenen Augeninnendruck Symptome. Dies mag an den multiplen Fehlerquellen der **Applantionstonometrie**

Applantionstonometrie

liegen [15], sodass falsch niedrig gemessene Druckwerte durchaus bei physiologischerweise dünnen Hornhäuten in Wirklichkeit intraokular höher sein können, so wie ein z. B. mit 6 mm Hg gemessener IOD bei einer zentralen Hornhautdicke von 459 µm und einer Kurvatur von nur 38 D etwa einem IOD von 10 mm Hg entspräche [16]. Andernfalls kann die Kataraktchirurgie den IOD signifikant falsch niedrig erscheinen lassen [17]. Ein derart voroperiertes Auge wird möglicherweise bei 4 mm Hg keine Zeichen einer Bulbushypotonie aufweisen. Andererseits muss auch beachtet werden, dass eine dicke Hornhaut nicht immer eine Korrektur des Druckwertes nach unten nach sich zieht, da bei einer ödematösen Hornhaut der Messkolben des Applanationstonometers auf der Hornhaut wie in einem weichen Schwamm einsinkt und somit die Druckmessung nach unten verfälscht [15, 17]. Die Messung wird durch die Hornhautdicke und die biomechanischen Eigenschaften der Hornhaut beeinflusst [18]. Der Intraokulardruck kann auch durch Palpation mit den Fingern durchs Lid abgeschätzt werden.

## Inzidenz der Bulbushypotonie nach Trauma

Die Inzidenz der **posttraumatischen Bulbushypotonie**

posttraumatischen Bulbushypotonie

ist unterschiedlich und hängt von der Art der Verletzung, dem Zeitintervall bis zur Vorstellung in der Klinik und der Art der primären Wundversorgung ab.

Bei einer **Bulbuskontusion**

Bulbuskontusion

ist die Hypotonia bulbi mit ca. 3 % angegeben [19]. Ist bei einer Kontusion oder Ruptur ein Glaskörperprolaps assoziiert, so finden sich in 43 % Irido- bzw. Zyklodialysen [19]. Nur 54–74 % der offenen Augenverletzungen (z. B. Bulbusberstung) sind initial hypoton [10].

## Anatomie und Physiologie

Physiologisch bedeutsam für die Aufrechterhaltung des IOD ist das Gleichgewicht zwischen der Produktion und dem Abfluss des **Kammerwassers**

Kammerwassers.

Etwa 7 % des gesamten Blutflusses des Auges passieren den Ziliarkörper. Dieser hat neben der Blut- und Nervenversorgung des vorderen Augenabschnittes auch die Aufgabe der Akkommodation, der Aufrechterhaltung der Blut-Kammerwasser-Schranke und der Kammerwasserproduktion [20]. Ionen und Wasser diffundieren passiv aus den gefensterten Ziliarkörperkapillaren in das Ziliarkörperstroma. Durch aktiven Natriumchloridtransport durch das Synzytium des Ziliarkörpers gelangen Wasser und Ionen in das pigmentierte Ziliarkörperepithel. Mithilfe der Na^+^/K^+^-ATPase und der Carboanhydrase II wird durch das nicht pigmentierte Ziliarepithel das Kammerwasser aktiv in die Hinterkammer sezerniert (Ultrafiltration) [13, 20, 21].

Dieser Prozess wird unter anderem von adrenergen Regelkreisläufen und dem Blutfluss durch den Ziliarkörper reguliert [20, 21]. Pro Minute werden etwa 2 µl Kammerwasser produziert [21]. Ein Vorderkammervolumen von etwa 200–300 µl würde in ca. 2 h 1‑mal komplett erneuert werden. Die Kammerwasserproduktion unterliegt auch zirkadianen Schwankungen: So ist sie tagsüber fast doppelt so hoch wie nachts im Schlaf [20, 21].

### Merke

Das Vorderkammervolumen wird ca. alle 2 h erneuert.

Der Abtransport des Kammerwassers erfolgt hauptsächlich über die folgenden **Abflusswege**

Abflusswege:

1. transtrabekulär und die episkleralen Venen sowie
2. uveoskleral, ein geringer Teil des Kammerwassers gelangt in die Hornhaut und wird via Endothelzellen zurück in die Vorderkammer gepumpt [13].

Traumatisch bedingt ist der Kammerwasserabfluss über eine Wunde [6].

Wird der neurosensorische Regelkreislauf von Kammerwasserproduktion und -abfluss gestört, kann auf der einen Seite ein Augenhochdruck mit der Gefahr einer Glaukomentwicklung und auf der anderen Seite eine Bulbushypotonie resultieren.

Wir unterscheiden 3 Arten der **posttraumatischen Bulbushypotonie**

posttraumatischen Bulbushypotonie:

1. akute Bulbushypotonie,
2. verzögerte Bulbushypotonie,
3. chronische Bulbushypotonie.

Im Folgenden wird auf die zeitlich unterschiedlichen Aspekte der Bulbushypotonie eingegangen.

### Akute Bulbushypotonie.

Die Bulbushypotonie kann akut nach dem Trauma durch den Parazenteseeffekt und den unkontrollierten Abfluss von Kammerwasser oder einen Glaskörperprolaps in die Wunde verursacht sein. Oberste Priorität haben bei **offener Bulbusverletzung**

offener Bulbusverletzung

[7] nach der Diagnostik (Computertomographie [CT]/Magnetresonanztomographie [MRT], Ultraschalluntersuchung, Spaltlampenbiomikroskopie) der Wundverschluss und, falls vorhanden, die Bergung eines intraokularen Fremdkörpers. Dies sollte zeitnah erfolgen [1, 10, 11, 22]. Bei **metallischem intraokularem Fremdkörper**

metallischem intraokularem Fremdkörper

ist eine MRT obsolet. Ein kleiner Parazenteseeffekt kann durch Abflachung der Vorderkammer auch zu einem Winkelblock mit sekundärem Druckanstieg infolge einer Tamponade der Wunde durch Iris oder Ziliarkörper führen. Ein verzögerter Wundverschluss kann zu einer traumatischen Epithelinvasion führen [10, 22, 23]. Stellt der Patient sich erst mehrere Stunden oder Tage nach der Verletzung vor, kann die Diagnosestellung der bulbuseröffnenden Verletzung erschwert sein, wenn der Druck durch eine Tamponade der Eintrittsstelle bzw. einen Winkelblock wieder angestiegen ist oder auch eine gedeckte Bulbusruptur vorliegt [1, 10]. Im Zweifelsfall ist die Exploration mit Peritomie empfehlenswert.

### Verzögerte Bulbushypotonie.

Schon nach Tagen kann verzögert durch Resorption eines Hyphämas und damit verbessertem Kammerwasserabfluss sowie durch den Intraokulardruck senkende Medikamente eine Bulbushypotonie induziert werden. Ebenso kann das Auge nach einer erfolgreichen primären Wundversorgung keinen Druck aufbauen, wenn ein angeschnittener oder abgerissener Ziliarkörper nicht direkt (unter Sicht durch die Sklerawunde chirurgisch fixiert wurde) [2]. Die Hypotonie nach Trauma kann auch medikamentös bedingt sein, was das Risiko einer posttraumatischen Nachblutung erhöht. Ein **traumaassoziiertes Hyphäma**

traumaassoziiertes Hyphäma

führt häufig zur Steigerung des intraokularen Drucks [5, 6, 8, 24]. Ein Black-ball- (die gesamte Vorderkammer ist mit schwarz gefärbtem, sauerstoffarmem Blut gefüllt) oder Red-ball-Hyphäma (die gesamte Vorderkammer ist mit Blut gefüllt und rot gefärbt) bedarf der intensiven medikamentösen oder auch entschlossenen chirurgischen Therapie [8], da neben der Sehnervenschädigung eine **Hämatokornea**

Hämatokornea

entstehen kann. Ein Black-ball-Hyphäma sollte spätestens am 4. Tag nach Trauma aus der Vorderkammer entfernt werden, ein Auge mit Red-ball-Hyphäma sollte spätestens am 6. Tag nach Verletzung gespült werden. Sinkt das Hyphäma (initial Grad IV = totales Hyphäma) nicht auf unter 50 % des Ausgangsniveaus am 6. Tag nach Trauma und persistiert ein Augeninnendruck über 25 mm Hg, so ist ebenfalls die Vorderkammerspülung erforderlich [8]. Bei großen Hyphämata sind aus diesem Grund tägliche Spaltlampenkontrollen erforderlich.

Gelingt die Resorption des Hyphämas und wirkt die drucksenkende Medikation nach, resultiert eine **passagere Bulbushypotonie**

passagere Bulbushypotonie.

Ein Absetzen bzw. eine Reduktion der augendrucksenkenden Medikamente erscheint sinnvoll.

### Chronische posttraumatische Bulbushypotonie

Ist über lange Zeit nach der Verletzung der Augeninnendruck zu niedrig (mehr als 4 Wochen), wird von einer chronischen posttraumatischen Bulbushypotonie gesprochen. Eine chronische posttraumatische Bulbushypotonie kann durch mehrere Komplikationen induziert worden sein: persistierende Ablatio retinae, Ziliarkörperinfarkt, Ziliarkörperkontusion, Ziliarkörperinflammation (z. B. bei sympathischer Ophthalmie), Zyklodialyse, Aderhautabhebung, Zilioschisis (Spaltung des Ziliarkörperepithels) oder eine Ringschwiele des Ziliarkörpers, anteriores posttraumatisches Fibrosesyndrom, PVR(proliferative vitreoretinale Retinopathie)-Membranen, anteriore Loop-Traktion, Kapselfibrose mit Zug auf die Ziliarkörperzotten [13, 25, 26, 27]. Die therapeutische Möglichkeit ist an der Ursache ausgerichtet – z. B.: ältere persistierende Netzhautablösung – Vitrektomie/Cerclage, Aderhautdefekt – Chorioidopexie, Kapselphimose mit Ziliarkörperzottenelongation bzw. beginnender Zilioschisis – Kapsulektomie oder radiäre Kapsulotomie, Zyklodialyse – Zykloplegie oder Zyklopexie.

Seltenere Ursache einer chronischen Bulbushypotonie können die Avulsio nervi optici, eine Endophthalmitis haemogranulomatosa bzw. eine Endophthalmitis phakoanaphylactica sein [8, 13].

Neben der PVR-Reaktion sowie Hornhautnarbenbildung führt auch die persistierende posttraumatische Bulbushypotonie zu einem **progredienten Sehverlust**

progredienten Sehverlust

[13]. Die komplette Rekonstruktion der Bulbuswand und die chirurgische Wiederanlage der Netzhaut nach bulbuseröffnender Verletzung allein reichen nicht aus, wenn das Auge danach hypoton bleibt und schrumpft. Schlimmstenfalls entsteht durch den Zug der 4 geraden Augenmuskeln bei weichem und geschrumpftem Auge der **Bulbus quadratus**

Bulbus quadratus

mit kosmetisch beeinträchtigenden Effekten und Schmerzen durch die Reizung der langen Ziliarnerven [13, 26].

Bei der Versorgung vieler Bulbusverletzungen fiel uns auf, dass der Misserfolg der Chirurgie oft mit einer Bulbushypotonie einherging. Etwa 3 % der Augen nach stumpfer Verletzung (Kontusion, Ruptur) waren weich aufgrund einer Zyklodialyse [24].

## Mechanismus Ziliarkörper-Kammerwinkeltrauma

### Kontusion

Bei einer Kontusion infolge einer stumpfen Verletzung wird der Augapfel sagittal komprimiert und durch die nachfolgende Entspannung elongiert [6, 19]. Dies kann Zerreißungen von Blutgefäßen, Trabekelmaschenwerk, Ziliarkörper, Iris, Zonulafasern, Glaskörperbasis, Netzhaut und Aderhaut zur Folge haben [6, 19, 27, 28]. Durch die Kontusionsverletzung kann im Kammerwinkel der Ziliarkörper von dem Skleralsporn abreißen – eine **Zyklodialyse**

Zyklodialyse

wäre die Folge [25, 29, 30, 31]. Dadurch fließt vermehrt Kammerwasser unter dem Ziliarkörper in den Suprachoroidalraum. Nicht nur eine Ziliarkörperabhebung, auch eine Aderhautabhebung kann resultieren, wodurch die Bulbushypotonie noch ausgeprägter wird [13]. Ist das Trauma so stark, dass die langen Ziliarkörperarterien abgeschert werden, kann der Ziliarkörper infarzieren. Dieser Infarkt ist ähnlich dem Aderhautinfarkt im Rahmen eines Hutchinson-Siegrist-Neubauer-Syndroms zu interpretieren [9, 24, 27]. Eine schwere PVR-Ablatio nach Contusio bulbi kann ebenfalls über eine sog. „anterior loop traction“ den Ziliarkörper abheben oder zu einer **Zilioschisis**

Zilioschisis

(Spaltung des nicht pigmentierten vom pigmentierten Ziliarkörperepithel) führen, wodurch die Kammerwasserproduktion drastisch reduziert wird [13].

### Bulbusruptur

Die Bulbusruptur kann in 2 Formen vorkommen: als offene oder als gedeckte Bulbusruptur [6, 7, 10, 32]. Dabei wird durch die Wucht eines Stoßes der IOD bis an das Limit der Geweberissfestigkeit pathologisch gesteigert. Im Augenblick des Einrisses der festen Bulbuswand (Hornhaut oder Sklera) sinkt der Augendruck drastisch ab. Der Bulbus reißt an den Prädilektionsstellen: Limbus, Ansätze der geraden Augenmuskeln, Eintritt des N. opticus und Operationszugänge, wie z. B. der Kataraktoperationstunnel oder das Interface nach Keratoplastik [2, 32, 33]. Die Identifikation der **Rupturstelle**

Rupturstelle

ist bei offener Ruptur zumeist relativ einfach. Die offene Bulbusruptur muss zeitnah chirurgisch geschlossen werden, um einer Epithelinvasion vorzubeugen. Bei durch Bindehaut oder Tenon gedeckter Bulbusruptur sind die zeitige Revision und Inspektion aller 4 Quadranten zwischen den geraden Augenmuskeln erforderlich, um die Rupturstelle zu finden und zu versorgen [1, 2, 10, 22, 23, 26].

### Penetrierende Bulbusverletzung

Durch die Eröffnung der Vorderkammer bei penetrierender Bulbusverletzung entsteht der **Parazenteseeffekt**

Parazenteseeffekt.

Typischerweise fließt über den Wundspalt Kammerwasser ab, und die Wunde wird durch Irisgewebe tamponiert. Die Irisinkarzeration dichtet meist den Wundspalt ab, wodurch der Augeninnendruck ansteigt. Durch die Manipulationen der Iris mittels pupillenwirksamer Medikamente kann sich der Wundspalt wieder öffnen. Verläuft der Perforationsweg durch Bindehaut, Sklera, Aderhaut und Netzhaut, fällt oftmals Glaskörper in den Wundspalt. Je nach Ausdehnung der Wunde können sowohl Netzhaut als auch uveales Gewebe inkarzeriert werden. Der in die Wunde inkarzerierte Glaskörper bildet eine Leitschiene für die Fistulation des Kammerwassers nach außen [1].

#### Cave

Auch bei weichem Traumaauge muss eine Fistulation ausgeschlossen werden!

### Perforierende Bulbusverletzung

Bei der perforierenden Bulbusverletzung existiert neben der Eintrittsstelle auch eine Austrittsstelle des Fremdkörpers [1, 2, 7]. Die Identifikation und Versorgung der Eintrittswunde allein sind hier nicht ausreichend. Der Parazenteseeffekt ist ähnlich der einer penetrierenden Verletzung [6, 25]. Zusätzlich verliert der Bulbus Kammerwasser oder Glaskörperflüssigkeit über die Austrittswunde. Der optimale Zeitpunkt der Versorgung der Austrittswunde am hinteren Pol wird derzeit noch kontrovers diskutiert. Eine **posteriore Versorgung**

posteriore Versorgung

der Austrittswunde ab interno bleibt dem erfahrenen vitreoretinalen Chirurgen vorbehalten [6, 11].

## Klinische Zeichen der Bulbushypotonie

Da der Augeninnendruck allein nicht für die Diagnose der klinisch signifikanten Bulbushypotonie ausreicht, sollte bei der **augenärztlichen Untersuchung**

augenärztlichen Untersuchung

und **Biomikroskopie**

**Figure 1:**
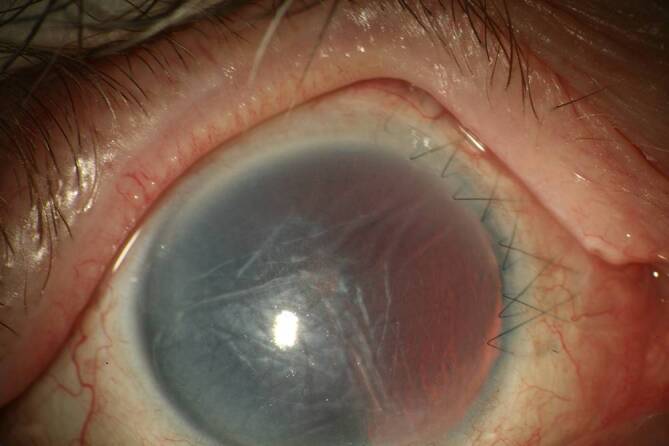

**Figure 2:**
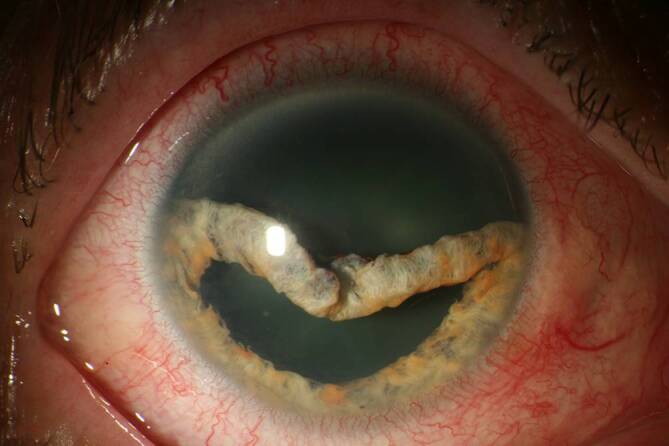

**Figure 3:**
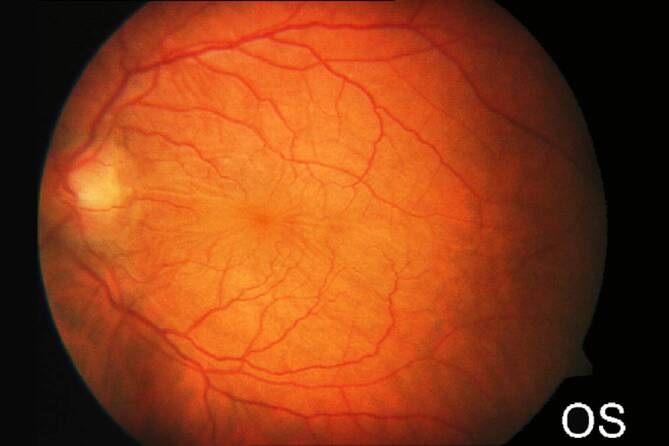

**Figure 4:**
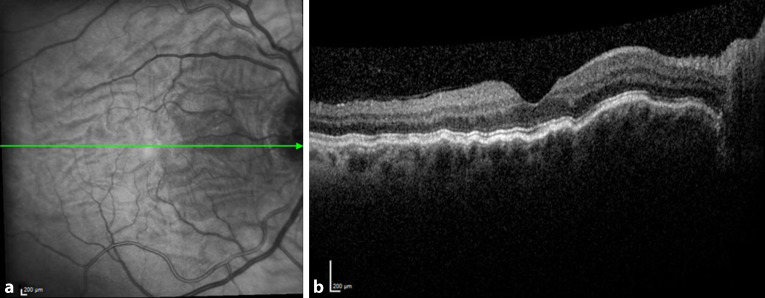

**Figure 5:**
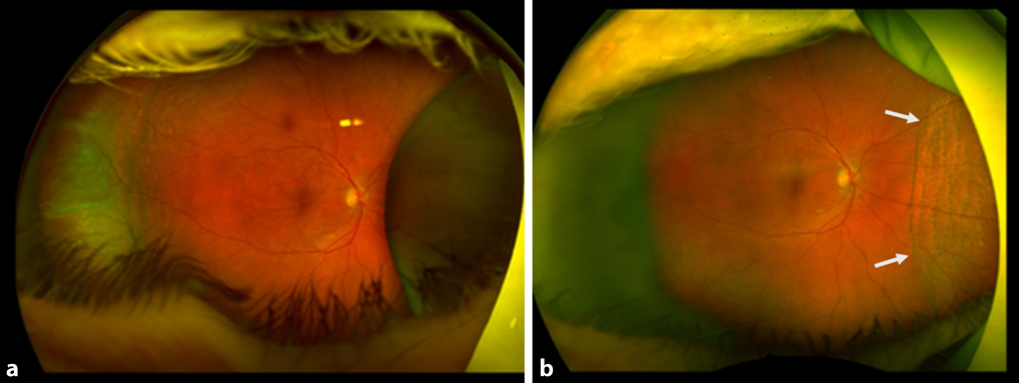

**Figure 6:**
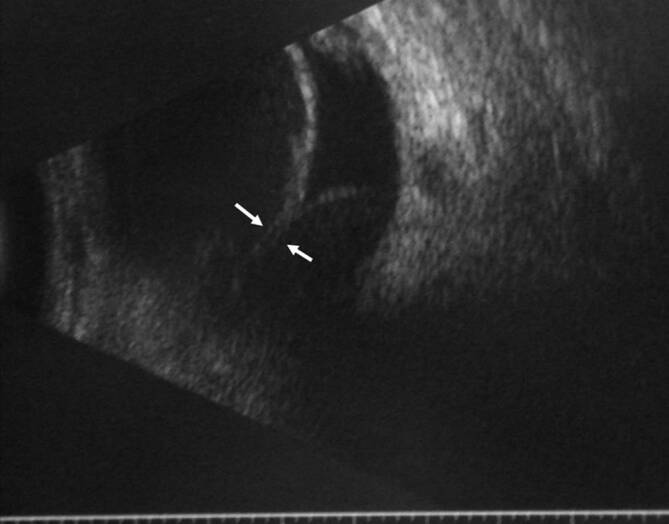

**Figure 7:**
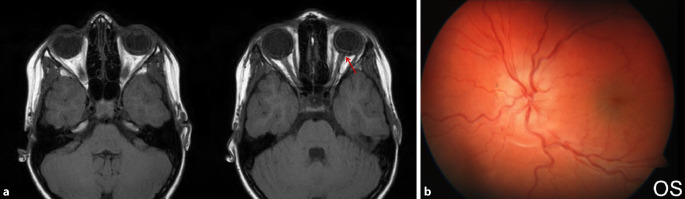

**Figure 8:**
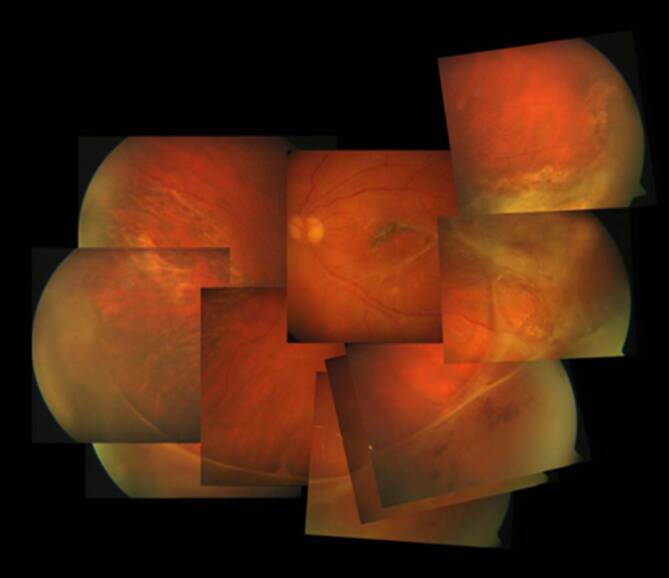

Biomikroskopie

nach einer geschlossenen Augenverletzung sowie nach primärer Wundversorgung einer offenen Bulbusverletzung äußerste Sorgfalt angewendet werden [6, 30, 31, 34, 35, 36]:

- Visus und Refraktion: Bei Bulbushypotonie schwillt die Aderhaut an, die Netzhaut erfährt einen Shift nach vorne, was allein zu einer Verkürzung der Achsenlänge Hornhaut-Netzhaut und somit zu einer Hyperopisierung führt. Andererseits kann durch einen Zonulafaserdefekt und eine nur kleine Zyklodialyse die Linse einen mehr kugelförmigen Zustand erreichen und das Licht stärker brechen, was zu einer Myopisierung bei Augen führen kann, die noch eine hohe Akkommodationsfähigkeit haben. Andererseits können ein segmentaler Zonuladefekt oder eine kleine Zyklodialyse zu einem ausgeprägten lentogenen Astigmatismus führen. Meist ist der Visus sine correctione herabgesetzt. Bei Kleinkindern muss diese Untersuchung besonders sorgfältig durchgeführt werden, da diese meist nicht über die Visusbeeinträchtigung klagen und sich unerkannt eine Amblyopie entwickeln kann.
- Hornhautfalten: Durch die Bulbushypotonie verliert die Vorderkammer ihr Volumen und die Sklera ihre Gewebespannung. Der limbale Skleraring ist nicht mehr straff gespannt. Die Descemet-Membran wird faltig, und die Hornhaut wird durch den geringen Druck partiell intransparent (Abb. 1). Später können in die dekompensierte und gefältelte Hornhaut stromale Blutgefäße einwachsen (Keratopathia striata).
- Vorderkammertiefe: Sowohl eine ungewöhnlich tiefe als auch eine ungewöhnlich flache Vorderkammer können mit einer Bulbushypotonie assoziiert sein. Eine sehr tiefe, aber auch sehr flache Vorderkammer nach Trauma entsteht z. B. durch Zonulaabriss, einen Kammerwinkelriss mit Verlagerung der Iriswurzel oder eine Linsen(sub)luxation. Eine im Seitenvergleich abgeflachte Vorderkammer kann z. B. durch eine Fistulation (Parazenteseeffekt, positive Seidel-Probe) aus der Vorderkammer oder durch Druck aus dem Glaskörperraum, z. B. durch eine ausgeprägte Glaskörperblutung oder eine Aderhautamotio resultieren. Ebenso kann durch eine Zyklodialyse der Ziliarkörper abgehoben sein und die Linse über ihre jetzt stärkere Kugelform die Vorderkammer abflachen. Liegt zusätzlich eine Iridodialyse vor, ist das Risiko für einen Ziliarkörperabriss erhöht (Abb. 2).
- Linsentrübung: Persistiert die Bulbushypotonie über Monate, wird infolge der gestörten Blut-Kammerwasser-Schranke der Eiweißgehalt im Kammerwasser höher, die Ernährung der Linse verändert sich drastisch, eine „Cataracta hypotonica“ ist die Folge.
- Netzhautfalten: Infolge der Hypotonia bulbi verlieren die Augenhüllen ihre Spannung, ähnlich wie ein faltiger Luftballon, wenn die meiste Luft aus ihm entwichen ist. Die sich der Aderhaut anschmiegende Netzhaut hat nun einen geringeren Anpressdruck aus dem Glaskörperraum, weshalb sie in Falten liegt. Die Anordnung der Netzhautfalten am hinteren Pol folgt oft der Schwerkraft – die Falten sind horizontal angeordnet – mit Ausnahme der Makula, hier kommen die radiären Leitschienen der Henle-Faserschicht zum Tragen, sodass Makulasternfalten resultieren (Abb. 3 und 4). Auch ein zystoides Makulaödem kann persistieren.
- Netzhautablösung: Bei Bulbushypotonie ist der Gegendruck zu den gefensterten Kapillaren der Choriokapillaris deutlich erniedrigt, eine exsudative Ablatio retinae kann im Spätstadium entstehen (Abb. 5).
- Aderhautfalten: Ist die Hypotonie nur mild ausgeprägt, schwillt die Aderhaut an und kann selbst (meist am hinteren Pol) insbesondere im Bereich der Makula – horizontale Falten aufwerfen (Abb. 4 und 5).
- Aderhautamotio: Bei exzessiv erniedrigtem Augeninnendruck entsteht die uveale Effusion, proteinreiches Transsudat tritt in den Suprachoroidalraum über. Die Aderhaut wölbt sich ballonartig über einen (Abb. 5) oder mehrere Quadranten in den Glaskörperraum vor. Wenn sich die gegenüberliegenden Aderhautanteile fast berühren, spricht man von „kissing choroids“ (Abb. 6).
- Papillenödem, Stauungspapille e vacuo: Durch den im Vergleich zum Liquordruck erniedrigten Augeninnendruck entsteht das Bild einer Stauungspapille (Abb. 7a, b).
- Epiretinale Membranen können sich ausbilden (Abb. 8).
- Sklerafalten: Eine lange persistierende Bulbushypotonie führt nicht allein zu Descemet-Falten, die Umbauprozesse betreffen auch die Sklera. Die Sklera nimmt an Dicke zu, der Sklera-zu-Sklera-Abstand sinkt, und durch den Zug der 4 geraden Augenmuskeln entwickelt sich ein Bulbus quadratus.
- Enophthalmus und Pseudoptosis: Das infolge der Bulbushypotonie verkürzte Auge erscheint nach hinten verlagert, die Lidspalte kann verengt sein.

## Diagnostik bei Bulbushypotonie

Die Diagnostik bei persistierender Bulbushypotonie umfasst bei inzwischen verschlossener Wunde die Visustestung mit und ohne Korrektur beider Augen, die Bestimmung der Akkommodationsbreite, die Spaltlampenbiomikroskopie, die Seidel-Probe, die Gonioskopie [6, 12, 28, 36], die Fundoskopie und Intraokulardruckmessung beider Augen, Achslängenmessung beidseits, Ultraschalluntersuchung, Ultraschallbiomikroskopie (UBM) bzw. Vorderabschnitts-OCT (optische Kohärenztomographie). Mitunter wird unnötigerweise eine zerebrale Magnetresonanztomographie(MRT)- oder Computertomographie(CT)-Untersuchung aufgrund der Papillenschwellung durchgeführt (Abb. 7a), um eine zerebrale Raumforderung auszuschließen, dies liegt jedoch eher an der fehlenden Diagnosestellung einer Stauungspapille e vacuo bei Zyklodialyse oder Bulbushypotonie. In jedem Fall sollte die augenärztliche Diagnostik abgeschlossen sein, bevor man eine aufwendige Bildgebung initiiert (eine Ausnahme stellen komplexe Orbita-Augen-Verletzungen oder der Ausschluss eines intraokularen Fremdkörpers dar). Im Rahmen **traumatologischer Fragestellungen**

traumatologischer Fragestellungen

hat die CT gegenüber der MRT den deutlich höheren Stellenwert, da hierdurch keine Gefährdung bei metallischen intraokularen Fremdkörpern vorliegt und zudem knöcherne Strukturen besser darstellbar sind.

### Cave

Eine MRT ist bei metallischem Intraokularfremdkörper obsolet!

Die **Gonioskopie**

Gonioskopie

kann den Zyklodialysespalt lokalisieren, ist jedoch erschwert, wenn zuvor mydriatische bzw. zykloplegische Augentropfen appliziert worden sind. Die Gonioskopie sollte möglichst bei spielender Pupille im Seitenvergleich durchgeführt werden. Bei flacher Vorderkammer kann mitunter die Zyklodialyse in der Gonioskopie nicht gesehen werden, hier kann intraoperativ die Vorderkammer mit Viskoelastikum vertieft werden, um den Zyklodialysespalt darzustellen.

Die **OCT-Untersuchung**

**Figure 9:**
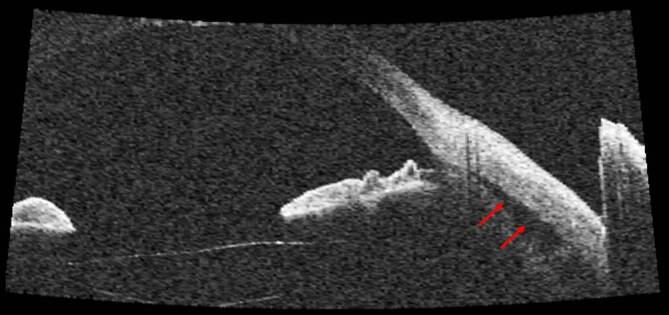

**Figure 10:**
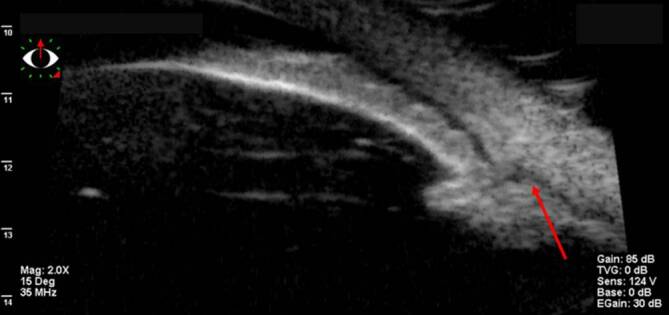

OCT-Untersuchung

zum Nachweis von Ziliarkörper- oder Aderhautveränderungen (Abb. 9) ist nur eingeschränkt nützlich [37]. Eine Zyklodialyse sowie pathologische Veränderungen im Bereich des Ziliarkörpers und Kammerwinkels lassen sich besser mit der UBM nachweisen (Abb. 10; [38, 8, 35, 37]). Bei mit Silikonöl gefülltem Bulbus und einem Zyklodialysespalt in der oberen Hemisphäre kann das Silikonöl in sitzender Position den Spalt verschließen und so die OCT-Untersuchung des vorderen Augenabschnitts verfälschen – hier empfiehlt sich die Untersuchung auch mit zur Seite geneigtem Kopf des Patienten.

### Cave

Silikonöl im Glaskörperraum erschwert die Erkennung eines Zyklodialysespaltes.

Für die **Ultraschallbiomikroskopie**

Ultraschallbiomikroskopie

bestehen prinzipiell 2 Möglichkeiten: Über einen auf den Bulbus gesetzten und mit Wasser gefüllten Trichter wird der UBM-Schallkopf an die Oberfläche angekoppelt. Die Alternative besteht in einem Kunststoffüberzieher (z. B. der Finger eines Operationsgummihandschuhs), der mit z. B. BSS (physiologische Kochsalzlösung) gefüllt ist und der mittels Kontaktgel (z. B. Hydroxymethylcellulose) an die Augenoberfläche angekoppelt wird. Dies ist auch transpalpebral möglich. Meist kommen 2 UBM-Schallköpfe für die Diagnostik zum Einsatz: ein 35-MHz- und ein 50-MHz-Schallkopf. Der 50-MHz-Schallkopf bietet eine höhere Auflösung, eine geringere Eindringtiefe und einen kleineren Bildausschnitt des Auges. Wir verwenden meist den 35-MHz-Schallkopf, weil damit ein größerer Augenausschnitt im Vorderabschnitt dargestellt werden kann.

## Therapie der Zyklodialyse

### Medikamentöse Therapie

**Steroide**

Steroide

zur Drucksteigerung zeigen bei der posttraumatischen Bulbushypotonie nur wenig Wirkung. Die Steroidresponse mit erheblichen Drucksteigerungen ist nur bei ca. 5 % der Bevölkerung (ca. 5 % bei emmetropen Augen, ca. 20 % bei myopen Augen) zu erwarten. Steroide können jedoch eine inflammatorische Hyposekretion von Kammerwasser positiv beeinflussen.

Bei kleineren und symptomatischen Zyklodialysen mit Bulbushypotonie [8, 25, 30, 31] – meist unter 2 Uhrzeiten – lohnt sich zumeist die **konservative Therapie**

konservative Therapie:

Zunächst wird mit lang wirksamen Mydriatika/Zykloplegika lokal therapiert (z. B. Cyclopentolat AT [Augentropfen] 4‑mal/Tag bzw. Atropin 1 % AT 2‑mal/Tag). Bei Kindern muss allerdings auf unerwünschte Nebeneffekte von Atropin hingewiesen werden (wir bevorzugen im Kindesalter Cyclopentolat AT).

#### Merke

Kleinere Zyklodialysen verschließen sich unter Zykloplegie.

### Chirurgische Therapie

Zeigt die Zykloplegie nach 3 Wochen noch keine Wirkung (keinen IOD-Anstieg), kann mittels einer **Laserkoagulation**

Laserkoagulation

(532 nm, cw) der skleraseitige Teil des Ziliarkörpers sanft koaguliert werden. Dieser Vorgang ist im Abstand von mehreren Wochen wiederholbar. Auch über Blutinjektionen in die Vorderkammer zum Verschluss der Zyklodialyse wurde berichtet [6, 8]. Kasuistisch wurde auch über Kapselspannringe berichtet, die in den Sulcus ciliaris platziert wurden. Die Eingabe von Viskoelastika in die Vorderkammer, um das Kammerwasser zäher zu gestalten und den Abfluss zu verlangsamen, hat sich bisher nicht durchgesetzt [8].

#### Zyklopexie

Steigt der Augendruck nicht an, ist die direkte Zyklopexie nach Naumann und Völcker zu erwägen [13, 23]. Eine simultane Operation, wie z. B. die Vitrektomie, die Linsenoperation oder die Irisplastik, ist möglich [2].

**Figure 11:**
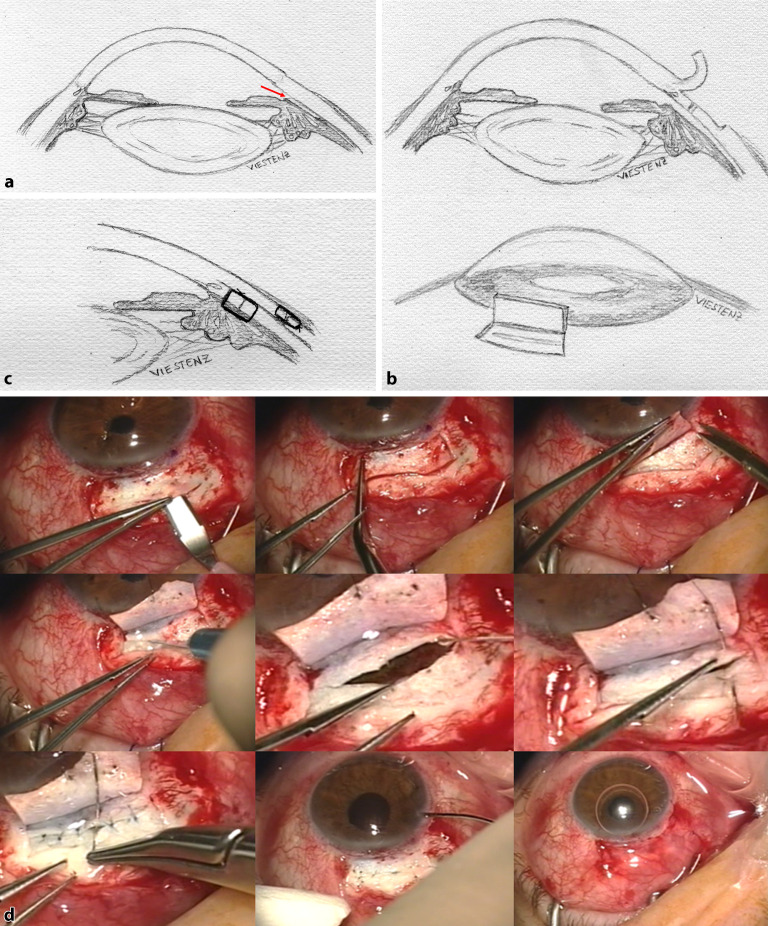

Die **direkte Zyklopexie**

direkte Zyklopexie

nach Naumann gilt noch immer bei geschlossenem Bulbus als Methode der Wahl [8, 13, 23, 29, 30], um bei hypotonem Bulbus, den abgerissenen Ziliarkörper an der Sklera zu refixieren. Hierfür ist eine genaue Lokalisation des Zyklodialysespaltes erforderlich. Es lohnt sich, bei Operationsbeginn zu gonioskopieren. Gelingt dies nicht, kann zuvor über eine Parazentese die Vorderkammer mit BSS gestellt werden. Dann wird auf der Sklera bzw. der Hornhaut die Ausdehnung der Zyklodialyse markiert. Alternativ lohnt sich kurz präoperativ im Liegen eine UBM-Untersuchung mit Markierung des Zyklodialysespaltes, da das Auge auch im Liegen zyklorotieren kann [39]. Nach der Peritomie wird ein Skleradeckel über dem Zyklodialysespalt von ca. 50 % Skleratiefe limbusständig präpariert und auf die Hornhaut umgeklappt. Danach wird in ca. 1–1,5 mm Limbusdistanz die verbliebene Sklera limbusparallel vorsichtig durchtrennt und der Ziliarkörper freigelegt. Nun kann der Ziliarkörper direkt mit einer gebogenen Nadel und Prolene 9‑0 wieder fixiert werden, oft sind auf eine Uhrzeit der Zyklodialyse 2 bis 3 radiäre transsklerale Einzelknüpfnähte (EKN) erforderlich. Nach der Zyklopexie wird der Skleradeckel darüber mit Nylon-10-0-EKN fixiert und die Bindehaut verschlossen (Abb. 11).

Sii und Agrawal empfehlen, präoperativ** Apraclonidin**

Apraclonidin

topisch zu applizieren, um die Ziliarkörperdurchblutung und damit hämorrhagische Komplikationen während der Zyklopexie zu minimieren [12, 40].

Agrawal und Krohn beschreiben die Methode der **Kryotherapie**

Kryotherapie

des Zyklodialysespalts [12, 41, 42]. Dabei wurde der Kryokoagulationsstab mit seiner Mitte 1,5 mm hinter dem Limbus über den Zyklodialysespalt positioniert und überlappende Kryoherde für 2–3 s bei −80 °C appliziert.

Auch die Kryotherapie, unterstützt von einer Vitrektomie mit Endotamponade des Glaskörperraums (z. B. expansives Gas) mit danach optimierter Lagerung des Patienten (Zyklodialyseareal oben) kann die Hypotonie kurieren [41].

Die Zyklopexie ist kombinierbar mit einer kompletten Rekonstruktion des Auges im Rahmen einer **Pol-zu-Pol-Chirurgie**

**Figure 12:**
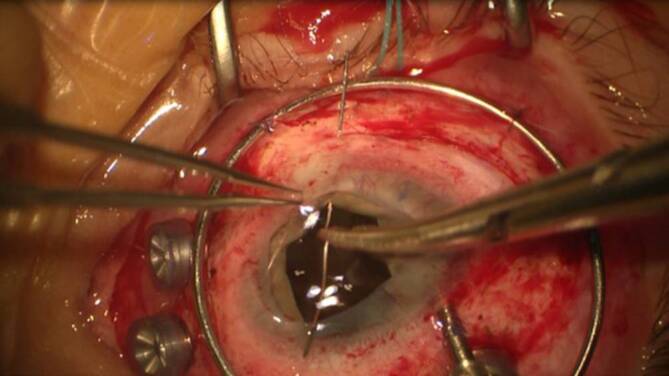

Pol-zu-Pol-Chirurgie

(perforierende Keratoplastik, Zyklopexie „open sky“ (Abb. 12), ggf. Irisplastik, Linsenoperation und Vitrektomie) [4]. Hierbei besteht die Möglichkeit, den Ziliarkörper von innen nach außen zu fixieren. Als Nahtmaterial wird Prolene 10‑0 oder Prolene 9‑0 verwendet. Im Gegensatz zu Nylon wird dieses nur sehr langsam zersetzt. Für eine langfristige Zyklopexie ist Vicryl nicht als Nahtmaterial geeignet.

## Postoperative Therapie nach Zyklopexie

Über mehrere Wochen (mindestens 3 Wochen) sollte die lokale Zykloplegie fortgesetzt werden. Steroide (z. B. Prednisolonacetat-AT) 5‑mal/Tag werden um 1 AT/Woche reduziert. Lokale Antibiotika (z. B. Ofloxacin AT 5‑mal/Tag und AS zur Nacht) werden 1 Woche nach Operation abgesetzt.

Wenige Tage nach der Zyklopexie kann es zu erheblichen Druckspitzen kommen, die prophylaktisch mit systemischen **Karboanhydrasehemmern**

Karboanhydrasehemmern

therapiert werden können. Agrawal berichtete in den ersten Tagen nach Zyklopexie über Druckanstiege von im Mittel ca. 40 mm Hg und im Einzelfall bis zu 70 mm Hg [12]. Unter einer systemischen und lokalen Drucksenkung sind diese IOD-Spitzen meist innerhalb von 1 Woche reversibel. Sie können jedoch extrem schmerzhaft für die Patienten sein, da z. B. ein Anstieg des Intraokulardrucks von 4 auf 40 mm Hg eine Verzehnfachung des Intraokulardrucks und einen erheblichen Reiz auf die Schmerzrezeptoren des Auges bedeutet. Eine zusätzliche **Schmerzmedikation**

Schmerzmedikation

sollte bereits am Operationsende angesetzt werden. Die Entwicklung von Sekundärglaukomen ist eher selten, kann aber zeitlich verzögert auftreten [6, 8].

### Cave

Nach operativer Zyklopexie sollten die Druckspitzen mit Karboanhydrasehemmern i.v. abgefangen werden!

### Okuläre Hypertension am kontralateralen Auge

Zu beachten ist, dass bei einer Bulbushypotonie das kontralaterale Auge konsekutiv mitreagiert. Durch die Hypotonie eines Auges erscheint im Regelkreislauf ein Signal an den Ziliarkörper beider Augen, mehr Kammerwasser zu produzieren. Das nicht von der Bulbushypotonie betroffene Auge kann eine okuläre Hypertension (OHT) entwickeln. Nach Druckanstieg des initial hypotonen Auges ist von uns oft eine Normalisierung des IOD des kontralateralen Auges beobachtet worden.

#### Cave

Die Bulbushypotonie kann eine OHT des kontralateralen Auges induzieren!

Steigt der Augeninnendruck des zyklopexierten Auges an, nimmt die Achslänge zu, die Akkommodationsbreite steigt bei phaken Augen, und der Visus kann sich bessern [12]. **Makulasternfalten**

Makulasternfalten

sind meist innerhalb eines halben Jahres weitgehend rückläufig.

### Sonderfall tiefer gedeckter Skleradefekt

Ist der Skleradefekt zu groß, kann z. B. ein **Tutopatch**

Tutopatch

auf die Sklera aufgebracht werden. Findet sich darunter ein Aderhaut-Sklera-Defekt, kann auch eine direkte **Chorioidopexie**

**Figure 13:**
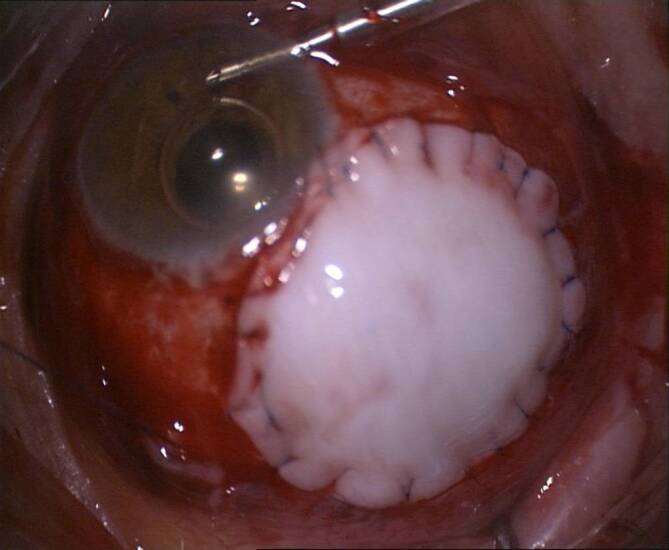

Chorioidopexie

mit nicht resorbierbarem Nahtmaterial durchgeführt werden (Abb. 13). Vorübergehend ist ebenso eine Tenondeckung [43] möglich oder eine Präparation eines invertierten Skleradeckels.

### Aderhautriss/-abhebung

Ursache einer Hypotonia bulbi kann weiterhin ein Aderhautabriss oder eine persistierende Abhebung der Chorioidea sein [2, 10, 13, 44]. Falls möglich, kann eine anterior oder äquatorial gerissene Aderhaut mit nicht resorbierbaren Fäden fixiert werden – eine andere Methode besteht in der Klebung der Aderhaut mit **Fibrinkleber**

Fibrinkleber

an die Sklera [45, 46, 47]. Hierbei muss aber auch beachtet werden, dass mit einsetzender Fibrinolyse und evtl. erneuter Bulbushypotonie die Aderhaut wieder abgehoben werden kann.

### Aderhautabhebung mit Netzhautablösung

Bei persistierender Aderhautabhebung mit Netzhautablösung lohnt sich eine **Vitrektomie**

Vitrektomie

mit langfristiger Silikonöltamponade. Augen mit einer höheren Myopie scheinen für die Kombination aus Aderhautabhebung und Netzhautablösung prädestiniert zu sein. Die Vitrektomieports sind bei hypotonem Auge/Ziliarkörper- bzw. Aderhautabhebung nicht einfach zu setzen.

#### Cave

Trokare bei Aderhautabhebung könnten die Infusion suprachoroidal einleiten!

Die **Trokare**

**Figure 14:**
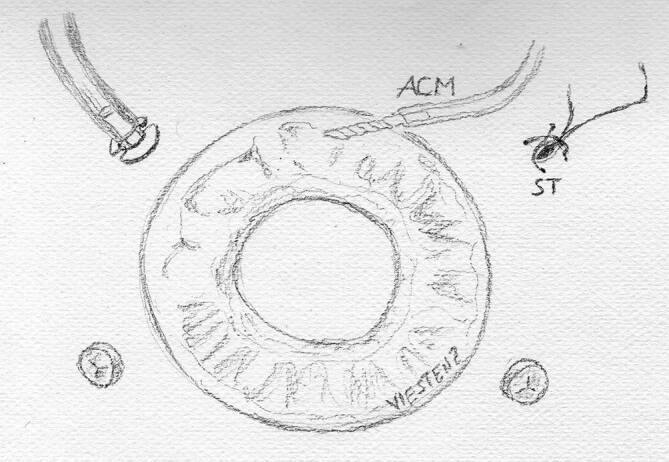

Trokare

könnten fälschlicherweise unter der Aderhaut oder dem Ziliarkörper positioniert werden und damit die Aderhaut‑/Ziliarkörperabhebung bei eingeschalteter Infusion an den Ports verstärken. Mehrere Möglichkeiten können hier Abhilfe verschaffen:

1. Über eine Parazentese wird der Augapfel durch Injektion von BSS oder Viskoelastikum in die Vorderkammer tonisiert. Dies birgt jedoch bei chirurgischer Manipulation am Augapfel das Risiko, dass Flüssigkeit aus der Vorderkammer ausgepresst wird.
2. Über eine schräg gestochene Parazentese wird ein „anterior chamber maintainer“ platziert, über den kontinuierlich BSS nachgeführt wird (Abb. 14).
3. Ein langer Infusionsport (z. B. 6 mm lang) wird mittels 20-G-Technik platziert.
4. Als Alternative zu 3. kann eine Sklerotomie angelegt werden und suprachoroidale bzw. supraziliare Flüssigkeit nach außen drainiert werden – dies gelingt meist bei angeschalteter Infusion am „anterior chamber maintainer“.

Danach kann die interne Rekonstruktion des Augapfels zur Wiederanlage der Netzhaut beginnen.

Nach Kuhn ist die permanente Silikonölfüllung des vorvitrektomierten Glaskörperraumes eine Möglichkeit, einer schweren Atrophia bulbi mit Schrumpfung zu begegnen. Allerdings heilt die **Silikonölendotamponade**

Silikonölendotamponade

nicht die Bulbushypotonie – sie füllt vielmehr den Glaskörperraum aus und kann zur Verlegung des Kammerwinkels oder zum Übertritt von Silikonöl in die Vorderkammer führen. Sollte das Auge aphak sein, ist hier die **Andoiridektomie**

Andoiridektomie

anzuraten: Bei leichtem Silikonöl sollte sie bei 6 Uhr und bei schwerem Silikonöl bei 12 Uhr angelegt werden, um einen – wenn auch geringen –Kammerwasserfluss zu ermöglichen [6]. Eine Stellung des Bulbus mit Viskoelastikum hat meist nur passagere Wirkung [6]. Bei persistierender Bulbushypotonie wurde die Option der Verlegung des Schlemm-Kanals mit einem verbliebenen Katheter, der sonst bei der Kanaloplastik Anwendung findet (pers. Kommunikation Dr. Wagner, Universität Magdeburg), beschrieben.

## „Minimal eye“ nach Trauma

Der **Ziliarkörper**

Ziliarkörper

ist für die Integrität des Augapfels von entscheidender Bedeutung, wie das Postulat von Prof. Naumann zum „minimal eye“ eindrucksvoll zeigt [26]:

- mindestens 4 mm klare Hornhaut mit transparenter optischer Achse,
- ein mindestens zur Hälfte offener Kammerwinkel mit intaktem Trabekelmaschenwerk,
- mindestens 30–50 % Residualfläche der Netzhaut,
- ca. 30–50 % der Axone des N. opticus,
- 210° intakte Pars plicata des Ziliarkörpers.

## Fazit für die Praxis

- Auch bei akuter posttraumatischer okulärer Hypotonie muss eine Eröffnung der Bulbuswand ausgeschlossen werden.
- Ziliarkörperveränderungen werden neben der Gonioskopie am besten mit der Ultraschallbiomikroskopie (UBM) erkannt.
- Eine über Monate anhaltende Bulbushypotonie führt zu irreversiblen Veränderungen und zur Schrumpfung des Auges.
- Bei großer Zyklodialyse über 2 Uhrzeiten ist die direkte Zyklopexie Methode der Wahl.
- Bei Bulbushypotonie sollte auch die konsekutive okuläre Hypertension des unverletzten anderen Auges behandelt werden.

## CME-Fragebogen

Wie viel Kammerwasser wird produziert?

Pro Stunde ca. 0,1 µl

Ein Viertel des Glaskörpervolumens pro Woche

In 1 h ca. 100 µl

Am Tag 7 ml

Genau so viel Volumen, wie abfließt.

Ein 6‑jähriges Mädchen sieht seit 3 Wochen auf dem linken Auge verschwommen (Visus RA [rechts Auge] sc 1,0 und Visus LA [linkes Auge] sc 0,1). Der Visus cc beträgt am linken Auge +3,5 sph −075 cyl/120° = 0,4. Es fallen eine vertiefte Vorderkammer, eine Lentodonesis und ein Papillenödem am linken Auge auf. Die rechte Papille ist vital und randscharf. Sie berichtet, vor ca. 3 Wochen beim Stockfechten im Gesicht getroffen worden zu sein. Wie gehen Sie weiter vor?

Sie führen eine Magnetresonanztomographie (MRT) durch – zum Ausschluss einer intrazerebralen Raumforderung.

Sie gonioskopieren bei Verdacht auf Zyklodialyse und starten eine zykloplegische Therapie bei einem Ziliarkörperabriss von 1,5 Uhrzeiten.

Sie untersuchen das Kind neurologisch bei Verdacht auf Foster-Kennedy-Syndrom.

Sie vermuten eine kontusionsbedingte Linsensubluxation und passen eine Kinderbrille mit Kunststoffgläsern an.

Sie operieren das Kind bei lentogenem Astigmatismus und Anisometropie mit einer Multifokallinse und einem Kapselspannring.

Wodurch kann die Bulbushypotonie verursacht werden?

Emulsifiziertes Silikonöl

Zilioschisis

Lentodonesis

Hämophthalmus

Zyklopexie

Was ist bei einer posttraumatischen Zyklodialyse von mehr als 3 Uhrzeiten eine vielversprechende therapeutische Option?

Für 4 Wochen den Befund zu beobachten

Bei erhöhtem Augeninnendruck am kontralateralen Auge eine filtrierende drucksenkende Operation durchzuführen

Eine Fibrinklebung des Ziliarkörpers vorzunehmen

Eine mydriatische Therapie für 3 Wochen zu initialisieren

Die direkte chirurgische Zyklopexie

Was können die klinischen Zeichen einer posttraumatischen Bulbushypotonie umfassen?

Akkommodationsstörung, horizontale Makulafalten

Cataracta syndermatotica, Stauungspapille e vacuo

Aderhautabhebung, geschrumpftes Auge, Pseudoptosis

Flache Vorderkammer, verdünnte Sklera

Lentogener Astigmatismus, Schnabel-Optikusatrophie

Ein 63-jähriger Schmied wurde von einem Metallteil am linken Auge getroffen. Das Auge ist weich, es besteht ein Hämophthalmus. Welches Vorgehen ist obsolet?

Sie prüfen den Status der Tetanusimmunisierung.

Sie decken das Auge vorsichtig ab.

Sie untersuchen den Patienten mittels Magnetresonanztomographie.

Sie planen die Bergung des potenziell intraokular lokalisierten Fremdkörpers und die primäre Wundversorgung zeitnah.

Sie führen eine Spaltlampenuntersuchung und Seidel-Probe durch.

Wodurch wird die chronische Bulbushypotonie verursacht?

Eine epiiridale Membran

Eine persistierende Zyklodialyse von weniger als 1 Uhrzeit

Eine Pigmentdispersion

Ein großes Retinektomieareal

Einen akuten Pupillarblock-Winkelblock

Was ist u. a. erforderlich, um eine residuale Funktionsfähigkeit des Augapfels zu gewährleisten?

Etwa 20 % der Netzhautfläche und eine klare Hornhaut müssen erhalten sein.

Etwa 210° Pars plana des Ziliarkörpers und 90 % offener Kammerwinkel sollten intakt sein.

Mindestens 7 Uhrzeiten des Ziliarkörpers mit sekretorischer Pars plicata sollten erhalten sein.

Etwa 10 % der Axone des Sehnervens und ein offener Kammerwinkel mit Endothelialisierung müssen vorliegen.

Ein Kammerwinkelriss über 8 Uhrzeiten und eine Silikonölfüllung des Glaskörperraumes gewährleisten die Funktionsfähigkeit des Auges.

Welche pathologische Veränderung sollte mit folgendem Verfahren therapiert werden?

Die Aderhautabhebung sollte mit einer Zyklopexie versorgt werden.

Eine Aderhaut- und Netzhautablösung sollten mit einer Retinektomie behandelt werden.

Bei einem Aderhautriss sollte der Glaskörperraum mit Silikonöl gefüllt werden.

Bei einer Zyklodialyse sollte die Aderhaut dauerhaft mit Fibrinkleber an der Sklera fixiert werden.

Bei einer Bulbushypotonie mit Kapselsackphimose kann die Kapsulotomie bzw. Kapsulektomie den Augeninnendruck anheben.

Wie oft tritt eine akute posttraumatische Bulbushypotonie auf?

Sie tritt in ca. 3 % nach einer Bulbuskontusion auf.

Sie ist in ca. 10 % Folge einer penetrierenden Bulbusverletzung.

Sie wird in ca. 15 % nach Bulbusperforation beobachtet.

Die akute Bulbushypotonie findet sich in 20 % bei offener Bulbusruptur.

Sie tritt in 15 % bei gedeckter Bulbusruptur auf.

## References

[1] Schrader W, Viestenz A (2008). Aktuelle Konzepte schwerer Bulbus eröffnender Verletzungen. Ophthalmologe.

[2] Viestenz A, Fiorentzis M, Seitz B (2017). Management offener Bulbusverletzungen. Klin Monatsbl Augenheilkd.

[3] Boiko E, Churashov SV, Haritonova NN, Budko AA (2013). Vitreoretinal surgery in the management of war-related open-globe injuries. Graefes Arch Clin Exp Ophthalmol.

[4] Gülmez M (2015). Mikrochirurgische Versorgung offener Bulbusverletzungen.

[5] Kuhn F, Masiak R, Mann L, Morris D, Witherspoon D, Kuhn F, Pieramici D (2002). The OTS: predicting the final vision in the injured eye. Ocular trauma: principles & practice.

[6] Kuhn F (2008). Ocular traumatology.

[7] Kuhn F, Morris R, Witherspoon CD, Heimann K, Jeffers JB, Treister G (1996). A standardized classification of ocular trauma. Ophthalmology.

[8] Viestenz A, Küchle M (2004). Stumpfes Augentrauma. Teil I: Stumpfes Vorderabschnittstrauma. Ophthalmologe.

[9] Viestenz A, Küchle M (2005). Stumpfes Augentrauma. Teil II. Stumpfes Hinterabschnittstrauma. Ophthalmologe.

[10] Viestenz A, Schrader W, Küchle M, Walter S, Behrens-Baumann W (2008). Management der Bulbusruptur. Ophthalmologe.

[11] Kuhn F, Schrader W (2018). Prophylactic chorioretinectomy for eye injuries with high proliferative-vitreoretinopathy risk. Clin Anat.

[12] Agrawal P, Shah P (2013). Long-term outcomes following the surgical repair of traumatic cyclodialysis cleft. Eye.

[13] Naumann GOH, Naumann GOH (1997). Glaukome und Hypotonie-Syndrome (Pathologie des abnormen intraokularen Drucks). Pathologie des Auges.

[14] Dellaporta A (1955). Fundus changes in postoperative hypotony. Am J Ophthalmol.

[15] Whitacre MM, Stein R (1993). Sources of error with use of Goldmann-type tonometers. Surv Ophthalmol.

[16] Orssengo GJ, Pye DC (1999). Determination of the true intraocular pressure and modulus of elasticity of the human cornea in vivo. Bull Math Biol.

[17] Heinrich MA, Eppig T, Langenbucher A, Walter S, Behrens-Baumann W, Viestenz A (2012). Comparison of Goldmann applanation and dynamic contour tonometry before and after cataract surgery. J Cataract Refract Surg.

[18] Kohlhaas M, Boehm AG, Spoerl E, Pürsten A, Grein HJ, Pillunat LE (2006). Effect of central corneal thickness, corneal curvature, and axial length on applanation tonometry. Arch Ophthalmol.

[19] Viestenz A, Küchle M (2001). Eine retrospektive Analyse von 417 Kontusionen und Bulbusrupturen und häufig vermeidbaren Unfallursachen: Das Erlanger Okuläre Contusions-Register (EOCR) 1985 bis 1995. Klin Monatsbl Augenheilkd.

[20] Forrester J, Dick A, McMenamin P, Lee W (1999). The eye. Basic sciences in practice.

[21] Bergua A (2017). Das menschliche Auge in Zahlen.

[22] Viestenz A, Seitz B, Viestenz A, Naumann GOH (2018). Epithelial invasion after open globe injury. Clin Anat.

[23] Naumann GOH, Völcker HE (1975). Block excision of intraocular processes. II. Epithelial ingrowth into the anterior segment of the eye. Klin Monatsbl Augenheilkd.

[24] Viestenz A (2004). Aderhautruptur nach Bulbuskontusion – eine Analyse anhand des Erlanger Okulären Contusions-Registers (EOCR). Klin Monatsbl Augenheilkd.

[25] Naumann GOH, Völcker HE (1981). Direkte Zyklopexie zur Behandlung des persistierenden Hypotonie-Syndroms infolge traumatischer Zyklodialyse. Klin Monatsbl Augenheilkd.

[26] Naumann GOH (2008). Applied Pathology for Ophthalmic microsurgeons.

[27] Siegrist A (1895). Traumatische Ruptur von Ciliararterien. Am Suisses Sc Med.

[28] Tönjum AM (1966). Gonioscopy in traumatic hyphema. Acta Ophthalmol.

[29] Küchle M, Naumann GOH (1990). Direkte Zyklopexie bei Zyklodialyse mit persistierendem Hypotonie-Syndrom. Fortschr Ophthalmol.

[30] Küchle M, Naumann GOH (1995). Direct cycloplexy for traumatic cyclodialysis with persisting hypotony; report in 29 consecutive patients. Ophthalmology.

[31] Ormerod LD, Baerveldt G, Sunalp MA, Riekhof FT (1991). Management of the hypotonous cyclodialysis cleft. Ophthalmology.

[32] Wenzel M, Aral H (2003). Gedeckte Bulbusruptur. Klin Monatsbl Augenheilkd.

[33] Rohrbach JM, Weidle EG, Steuhl KP, Meilinger S, Pleyer U (1996). Traumatic wound dehiscence after penetrating keratoplasty. Acta Ophthalmol Scand.

[34] Costa VP, Arcieri ES (2007). Hypotony maculopathy. Acta Ophthalmol Scand.

[35] Hwang JM, Ahn K, Kim C, Park KA, Kee C (2008). Ultrasound biomicroscopic evaluation of cyclodialysis before and after direct cyclopexy. Arch Ophthalmol.

[36] Shaffer RN, Weiss DL (1962). Concerning cyclodialysis and hypotony. Arch Ophthalmol.

[37] Eppig T, Gillner M, Langenbucher A, Seitz B, Viestenz A (2011). Contact free in-vivo imaging of cornea and anterior chamber of the human eye—a qualitative comparison of imaging techniques. Klin Monatsbl Augenheilkd.

[38] Viestenz A, Seitz B, Deland E, Fiorentzis M, Latta A, Käsmann-Kellner B (2018). Clinical anatomy of the anterior chamber angle in congenital aniridia and consequences for trabeculotomy/cyclophotocoagulation. Clin Anat.

[39] Viestenz A, Langenbucher A, Seitz B, Viestenz A (2006). Impact of eye’s cyclorotation and axial orientation analysis of toric intraocular lenses: recommendations for an optimized evaluation of rotational stability of toric IOLs. Klin Monatsbl Augenheilkd.

[40] Sii F, Todd B, Shah P, Chiang M (2006). Reduction of anterior segment vascularity with preoperative topical apraclonidine 1. J Cataract Refract Surg.

[41] Hoerauf H, Roider J, Laqua H (1999). Treatment of traumatic cyclodialysis with vitrectomy, cryotherapy, and gas tamponade. J Cataract Refract Surg.

[42] Krohn J (1997). Cryotherapy in the treatment of cyclodialysis cleft induced hypotony. Acta Ophthalmol Scand.

[43] Fries FN, Suffo S, Daas L, Seitz B, Fiorentzis M, Viestenz A (2018). Tenonplasty for closing defects during sclerocorneal surgery—a brief review of its anatomy and clinical applications. Clin Anat.

[44] Bordeianu CD (1984). The appropriateness of our therapeutic attitude to expulsive hemorrhage. J Fr Ophtalmol.

[45] Coleman DJ, Lucas BC, Fleischman JA, Dennis PH, Chang S, Iwamoto T, Nalbandian RM (1988). A biologic tissue adhesive for vitreoretinal surgery. Retina.

[46] Jiang YR, Tao J, Jonas JB (2010) Traumatic choriodialysis treated by intraocular fibrine glue. Acta Ophthalmol Scand 88(4):e129–130. 10.1111/j.1755-3768.2009.01542.x 10.1111/j.1755-3768.2009.01542.x 19493252

[47] Silver FH, Wang MC, Pins GD (1995). Preparation and use of fibrin glue in surgery. Biomaterials.

